# Using a Polygenic Score to Predict the Risk of Developing Primary Osteoporosis

**DOI:** 10.3390/ijms231710021

**Published:** 2022-09-02

**Authors:** Bulat Yalaev, Anton Tyurin, Inga Prokopenko, Aleksandra Karunas, Elza Khusnutdinova, Rita Khusainova

**Affiliations:** 1 Laboratory of Human Molecular Genetics, Institute of Biochemistry and Genetics-Subdivision of the Ufa Federal Research Centre of the Russian Academy of Sciences, 450054 Ufa, Russia; 2 Internal Medicine Department, Bashkir State Medical University, 450008 Ufa, Russia; 3 Section of Statistical Multi-Omics, Department of Clinical and Experimental Medicine, School of Biosciences and Medicine, University of Surrey, Guildford GU2 7XH, UK; 4 Department of Genetics and Fundamental Medicine, Bashkir State University, 450008 Ufa, Russia; 5 Department of Medical Genetics and Fundamental Medicine, Bashkir State Medical University, 450008 Ufa, Russia

**Keywords:** osteoporosis, polygenic risk score, fracture, low bone mineral density

## Abstract

Osteoporosis (OP) is a multifactorial bone disease belonging to the metabolic osteopathies group. Using the polygenic score (PGS) approach, we combined the effects of bone mineral density (BMD) DNA loci, affecting osteoporosis pathogenesis, based on GEFOS/GENOMOS consortium GWAS meta-analysis. We developed models to predict the risk of low fractures in women from the Volga-Ural region of Russia with efficacy of 74% (AUC = 0.740; OR (95% CI) = 2.9 (2.353–3.536)), as well as the formation of low BMD with efficacy of 79% (AUC = 0.790; OR (95% CI) = 3.94 (2.993–5.337)). In addition, we propose a model that predicts fracture risk and low BMD in a comorbid condition with 85% accuracy (AUC = 0.850; OR (95% CI) = 6.6 (4.411–10.608)) in postmenopausal women.

## 1. Introduction

Osteoporosis is a systemic skeletal disease characterised by an increased risk of bone fractures, reduced bone mineral density (BMD) and disruption of the microarchitecture of cancellous and cortical bone [1]. The principal reason for the development of these processes is disruption of bone metabolism, in which catabolism and bone resorption begin to prevail over osteoblastogenesis, osteogenesis and bone mineralisation [2]. Primary osteoporosis includes postmenopausal, senile, juvenile and idiopathic types [3]. Secondary osteoporosis is associated with a number of comorbidities or with taking medication that can have a negative effect on bone remodelling [4]. It is estimated that 33% of women and 20% of men are at risk of developing an osteoporotic fracture, particularly in the hip, spine and forearm [5,6]. The WHO (World Health Organization) operational definition of osteoporosis is based on a DXA measurement of bone mineral density, based on the clear link between lower BMD and increased fracture risk [7,8].

Three groups of drugs are used to treat OP [9,10]: stimulants of bone matrix formation, inhibitors of bone resorption, dual-action drugs. However, despite effective therapy for OP, early diagnosis of the disease remains a concern. There has been no reduction in fracture frequency and the number of patients with diagnosed osteoporosis is increasing worldwide, despite the availability of Garvan, FRAX and QFracture 10-year probability calculators [11,12,13]. In replicative studies, the AUC (Area Under Curve) of the Garvan and FRAX models ranged from 0.61 to 0.85, with a mean value of ~0.7. One would want the predictive power to be greater than 80%. Diagnostic systems for osteoporosis with better predictive potential need to be developed, e.g., using genetic studies [11].

The GEFOS/GENOMOS consortium on genome-wide search for genetic markers of primary osteoporosis identified 56 loci associated with low bone mineral density, 14 of which were associated with the risk of osteoporotic fractures [8]. These results resulted in an unexpected conclusion: fractures and low BMD may be independent endophenotypes of OP, which changes the fundamental ideas regarding the aetiology of the disease and requires new approaches to the diagnosis and therapy of OP [14]. However, the results of GWAS replication within this consortium, as well as in a number of other independent studies have not reproduced many significant associations [15]. This is particularly well demonstrated by the data for a sample of women from the Volga-Ural region of Russia (VUR) [8]. In the summary data of genetic effects and their confidence intervals, the most significant DNA variants from the population of European and Oriental origin were not the main risk contributors for a sample of women with OP from the Volga-Ural region [8,15]. VUR is an ethnically diverse region on the border between Europe and Asia near Russia’s Ural Mountains. That is home to Finno-Ugric, Turkic and Slavic peoples, who have European and Mongoloid components in their gene pool in various proportions. The results of population research in this region agree with the data demonstrating the largest European contribution to the gene pool of the Mordvins, Komi and Russians and an increased Mongoloid component in the Turkic populations of the VUR [16].

This indicates the heterogeneous genetic structure of osteoporosis and the superior contribution of the ethnic component to the molecular pathogenesis of the disease. This actualises the definition of the DNA markers with the largest effect on osteoporosis and recognises that the implementation of advanced methods of complex statistical analyses is even more significant when studying multifactorial diseases in populations with a unique gene pool and ethnic structure, as compared to Western European populations [14]. Thus, there is a specific knowledge gap in both understanding the contribution of genetic factors to the risk of osteoporosis in ethnically differentiated populations and in understanding how the findings from GWAS and gene-candidate studies can be applied to improve the prognostic power of the current early osteoporosis diagnosis system.

One method employed to assess the contribution of a large number of loci to disease risk is the polygenic score (PGS) approach. In this method, the effects of the associated variant risk alleles (OR/beta) are combined into a PGS, reflecting part of an individual susceptibility to disease in a population under study. More specifically, the PGS is based on the sum of the effects of independent risk variants associated with the disease, using current data from the largest and most informative genome-wide association studies [17, 18]. Most studies in this direction are related to cardiovascular diseases, type 2 diabetes, breast and prostate cancer, as well as Alzheimer’s disease [19]. Simultaneously, there are only a few studies dedicated to the polygenic risk definition of osteoporosis [20,21]. In clinical practice, there is a growing need to implement PGS for early diagnosis of OP prior to the occurrence of fractures [8]. The polygenic risk method was first successfully applied in the GWAS study of schizophrenia. In this study, PGS explained approximately 3% of the variance in schizophrenia [22]. Currently, this method is used to predict the biological properties of crops [23] and livestock [24], and to assess the risk of disease in humans [17]. Currently, the most convincing evidence of the effectiveness of PGS in response to treatment is the use of statins to reduce the risk of a first coronary event, as studies have confirmed that the relative risk reduction is higher in individuals with a high genetic risk of cardiovascular disease [25,26]. Large samples of unrelated individuals in human populations and random gene drift allow this method of analysis to be employed more efficiently; however, it also requires careful quality control of the data studied [23,27]. The use of PGS for the development of prognostic models of fractures and low BMD separately and combined, as well as the search for the most sensitive and specific risk models for primary osteoporosis appear to be relevant.

The aim of this study is to assess the polygenic risk of fractures and low BMD based on the analysis of 140 polymorphic loci of microRNA target sites, polymorphic variants of candidate genes, along with genes associated with primary osteoporosis. We focused our research on the GWAS results for women with postmenopausal osteoporosis.

## 2. Results

**Figure 1:**
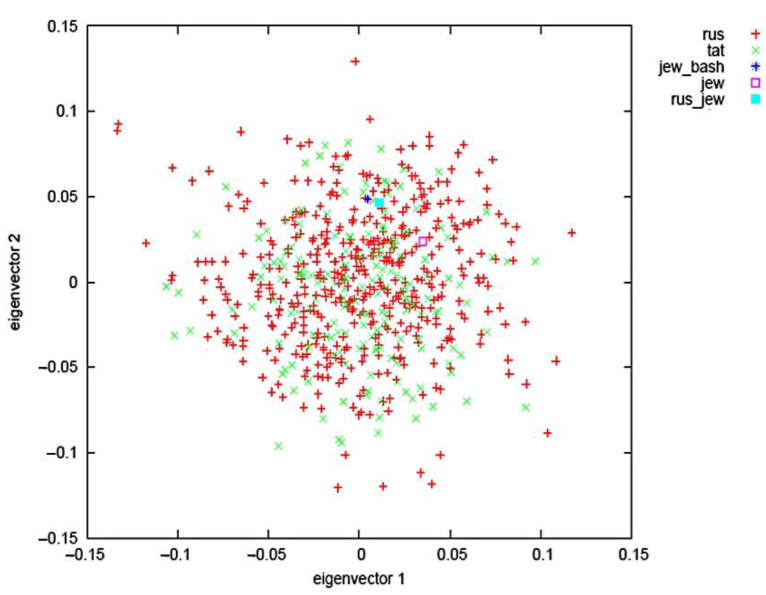
Location of women from the VUR for two principal components defined by Eigen vectors and the ethnic origin.

To determine whether we would need to account for the population substructure within the studied sample of women, who predominantly self-reported as being of Tatar and Russian descent, we conducted principal component analysis (PCA), by using the information from the independent loci studied. Individuals with a 97% success rate of genotyping and SNPs with a 97% success rate of genotyping were included in the analysis. In general, no population stratification of the studied sample by the studied loci depending on their ethnicity was observed (Figure 1).

Overall, the sample of women was not genetically heterogeneous, which supported our analysis of polygenic risk assessment of fractures and low BMD without dividing into subgroups by ethnic origin, i.e., the analysis was performed as for a single population. The QC, involving the exclusion of loci that were in linkage disequilibrium and do not support the Hardi-Weinberg equilibrium, resulted in 140 loci out of a total of 150 loci being tested. The range of minor allele frequencies of these loci ranged from 1% to 49%, which was consistent with the required quality control requirements for PGS.

### 2.1. Model for Predicting Fracture Risk in Women

**Figure 2:**
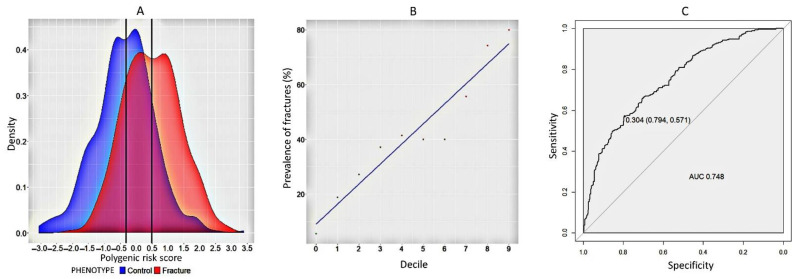
(**A**) The graph of the density distribution of polygenic risk score in the control and cases in general; (**B**) decile plots by PGS within each decile for cases (fracture) and controls in women in the general sample; (**C**) ROC-curve relating to the PGS of fractures in women participating in this study.

We calculated the PGS for each individual and assessed the relationship between PGS and osteoporotic fracture. We plotted the density distribution of indicators between the control and experimental groups (Figure 2A). The overwhelming proportion of polygenic indicators in women with fractures (median = 0.494) exceeded those in women without fractures (median = 0.357). The decile plot dividing the polygenic scale into 10% groups reveals that the polygenic risk index increases linearly with increasing fracture frequency (Figure 2B). ROC analysis demonstrated a high AUC (95% CI = 0.75 (0.71–0.78), sensitivity = 0.79 and specificity = 0.57 of the model (Figure 2C). Association analysis was statistically significant and indicated that the probability of fracture in the experimental group was 2.9 times higher (95% CI = 2.35–3.53), *p*-value = 2.37 × 10^−16^) compared to the control group.

### 2.2. Model to Predict the Formation of Low BMD in Women

**Figure 3:**
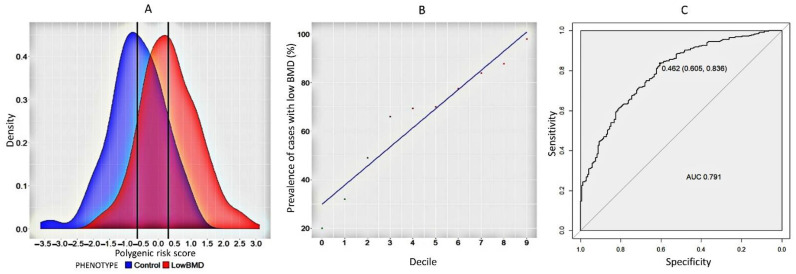
(**A**) The graph of the distribution of the density of polygenic risk score for the formation of a low level of BMD in the control (blue) and cases (red) in general; (**B**) decile plots by PGS within each decile for cases (low BMD) and controls in women in the general. (**C**) ROC-curve analysis of the polygenic risk assessment of the formation of a low level of BMD in women in the general sample.

According to the GEFOS/GENOMOS full genome study, it was determined that low BMD can be a separate endophenotype of osteoporosis, independent of osteoporotic fractures [8]. Therefore, it is of interest to study the contribution of the polygenic risk of this phenotype to OP. The analysis proved that the probability of forming a low BMD level was 3.94 times higher in the group with low mineral density compared to controls (95% CI = 2.99–5.34). To assess the difference in the distribution of the polygenic scores between the case and control groups, a plot of the density distribution of the scores between the control and experimental groups was plotted using R software (Boston, MA, US) (Figure 3A). As can be noted from the graph, the overwhelming proportion of polygenic scores in women with low BMD (mean value = 0.35) exceeded those in women with normal BMD (mean value = −0.65). In addition, we plotted the deciles of polygenic scores as a function of fracture incidence (Figure 3B). We report relatively high AUC = 0.791, sensitivity = 0.605 and specificity = 0.836 of the model (*p*-value = 2.54 × 10^−16^, Figure 3C).

### 2.3. A Model to Predict Fractures and Low BMD in Comorbid Women

**Figure 4:**
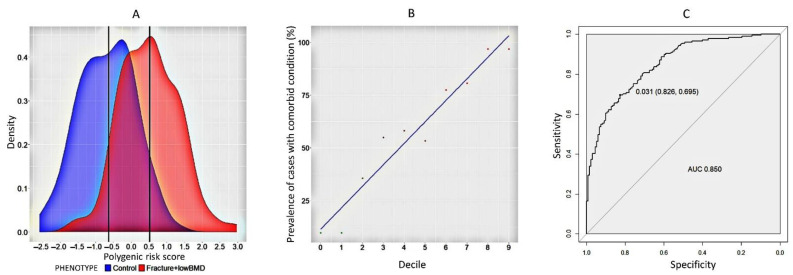
(**A**) The graph of the distribution of the density of PGSs of fracture with a low level of BMD in the control group and cases in women; (**B**) decile plots by PGS within each decile for cases and controls in women in the general; (**C**) ROC-curve analysis of PGS of fractures with a low level of BMD in women in the general sample.

Osteoporosis is a combination of two phenotypes–fractures and low BMD. Therefore, we developed a prognostic polygenic risk model in which women with both traits in a comorbid condition were analysed. At this stage, the study sample included patients with per-fractures and low BMD, therefore, a separate analysis of these cases was performed to determine the risk of these combined conditions. As can be established from the density distribution plot of the polygenic risk scale, the polygenic score of women with low BMD and fractures (median value 0.511) in the combined condition is higher than that of women with normal BMD and no fractures (median value −0.686) (Figure 4A). The decile plot illustrates a linear increase in the incidence of cases with the combined phenotype (Figure 4B). Sensitivity and specificity analysis of the model shows a high AUC (95% CI = 0.850 0.809–0.892), sensitivity = 0.826) and specificity = 0.695) (Figure 4C). Association analysis of the model confirmed a 6.6-fold (95% CI: 4.411–10.608) higher probability of fracture compared with the control group (*p* = 2.76 × 10^−16^). The model has the highest prognostic potential in the total sample of women.

## 3. Discussion

Based on the results obtained in this work together with the GWAS replication data within the GEFOS consortium, we first attempted to develop PGS models that predict low BMD and risk of osteoporotic fractures separately and in a comorbid condition in women from the VUR. Overall, our study findings are in each resulting model, the top percentile accounted for people at high risk of low-traumatic fractures and low BMD. It was determined that all the constructed models have high prognostic significance, allowing for a high degree of probability to correctly classify patients and the control group, as well as correctly, based on numerical indicators, differentiate differences on a polygenic risk scale. The highest indicators of sensitivity and specificity were exhibited by models of fracture risk and low BMD in combined conditions (AUC > 0.800).

Comparison with previous studies and the research value of our results. These results are new and interesting, given that the predictive models obtained in previous studies had less predictive value. For example, the use of the Lasso algorithm to construct a genetic risk scale in Li et al. (2019), established that the AUC area reduced from 0.653 to 0.587 and 0.588 in models that included clinical factors contributing to lower femoral neck BMD [28]. The authors emphasised the limited effectiveness of current genetic studies that focus on people of European descent when trying to improve the prediction of multifactorial disease and called for increasingly extensive studies targeting other ethnic groups [28]. Using data from the British Biobank, Tianyuan Lu et al. (2021), developed a polygenic risk scale of 21,717 genetic variants (r^2^ = 23.2%) explaining 25% of the total fracture risk variance [20]. This significantly exceeded the previous estimate of 22,886 SNPs, which explained 17.2% of the total variance and achieved an AUC value of 0.570 when studying patients with any type of fracture [21]. In comparison, the clinical risk cofactors FRAX, age, sex and their combination explained only 4% of the polygenic risk variance. PGS has been shown to be independent of other commonly used clinical risk factors and to be significantly associated with fracture risk [29]. However, it is recognised that the polygenic risk markers relevant to populations of European origin are not quite effective for prognostic models of fractures and the formation of low BMD in Chinese populations.

According to our data, low-traumatic fractures occur on the upper percentile, which is consistent with the results obtained by Tian-Yuan Lu et al. (2021), who explained that individuals with common fractures belonged to the group below the third percentile (2.5%) (95% ≈ 1.5–3.6%). Simultaneously, the incidence of severe osteoporotic fractures and hip fractures was only 0.7% (95% CI 0.2–1.4%) and 0.3% (95% CI 0–0.7%), respectively, among individuals with a PGS above the 99th percentile. Thus, polygenic risk assessment effectively predicts cumulative fracture risk, with low-traumatic fractures characterised by an increased polygenic risk [20]. Kim (2018) analysed a cohort from the British Biobank, in which a PGS of low BMD formation was performed based on LASSO analysis. There was a tendency for the risk of osteoporosis to decrease dramatically with increasing LASSO scores. For example, those with a low LASSO score of 4 (2.2% of individuals) had a 19-fold higher risk of developing OP than the middle cell (95% CI (6.18–6.20)). Those with the highest risk of OP were 0.221 (95% CI (0.218–0.224)) relative to the median [21].

The results of the HoLe et al. (2016) study, which genetically assessed the risk of per-relapse fractures and decreased BMD of the femoral neck and lumbar spine, are of interest. Based on data on 62 polymorphic variants, the authors revealed that the probability of fracture was significantly higher in men and women when the polygenic risk value was >4.24. In the Cox Proportional Hazards Model, an increase in polygenic risk of decreased femoral neck BMD was associated with a 20% increase in fracture risk. In addition, analysis by fracture type showed that the relationship between femoral neck polygenic risk and fracture risk was observed for spine and wrist fractures, but not for femoral neck fractures. The magnitude of the relationship remained almost unchanged after adjustment for age, sex, previous fracture and number of falls in the previous 12 months. However, after an additional correction of the BMD level of the femoral neck, the association between the polygenic risk of decreased BMD and fractures in general was no longer statistically significant [30]. The small effect size of polymorphic variants is to be expected according to a simple epidemiological principle [31]. Research data suggest that with an OR of up to 1.2 and assuming a 5-year per-relapse rate of 10%, the area under the ROC curve associated with this genetic variant is expected to be about 0.55. Thus, a single risk allele alone would not be helpful in predicting the risk of OP. At the same time, a combination of several genetic variants may be useful even for modest effect sizes, because the discriminatory power of the AUC increases in proportion to the effect size [32]. For example, when summing the effects of 50 genetic variants with an OR = 1.1 size, the AUC would be roughly 0.63; with an OR = 1.2, the AUC is expected to be about 0.73. It is assumed that a model with 500 variants, each with an OR = 1.1, could result in an AUC of up to 0.80.

Polygenic risk assessment is designed to improve the quality of predictive medicine [33], although its results should be interpreted with extreme caution, taking into account the ethnic origin of the sample and the variety of bioinformatic approaches to statistical analysis [34]. Thus, the results of PGS can be valuable in diagnostic medicine and have higher prognostic power at the individual level. Nonetheless, previously obtained results have low prognostic power, which generates considerable debate as regards their direct clinical benefit [35]. Consequently, it is necessary to continue research in this way, as the clinical implementation of prognostic methods for osteoporosis can be beneficial in cases where there is a higher a priori probability of disease, for example, in the early stages of the disease, to help in the diagnosis or to inform the choice of treatment [17].

Clinical value of the data obtained. The development of prognostically accurate models of osteoporosis genetics depends less on the coverage of the number of loci studied and the power of statistical research parameters but rather on studying the relationship between loci and OP phenotypes in individual bone localisations, which allows us to take a narrower approach to the tactics of osteoporosis research. In terms of finding risk markers, it is interesting to study the weighted effect of many markers on an individual’s risk of developing the disease, which can be measured using polygenic risk assessment. The efficiency of osteoporosis prognosis using polygenic assessment has increased. This is probably due to the absence of measurement error inherent in the conventional risk calculation using FRAX. In addition, PGS is independent of other clinical FRAX risk factors, and finally, genotypes are measured with very high accuracy. It is currently undetermined which bioinformatic analysis methods are best suited for predicting osteoporosis. There is a high contribution of the ethnic component to the genetic factor in the development of osteoporosis; in addition, indicators can be influenced by different proportions of heritable ethnic components, which can lead to a lower proportion of sensitivity models in a sample of women. Since allele and genotype frequencies can vary between ethnic groups, each allele can be at one frequency in one ethnic group but less frequent in another. Consequently, the effect size (e.g., odds ratio) for a risk allele may differ for different ethnic groups, particularly given different environmental factor backgrounds. Population characteristics (such as size, founder influence and inbreeding), as well as inheritance patterns may also differ. Thus, a polygenic risk assessment constructed when studying one ethnic group may not be applicable to another. To date, most PGS osteoporosis studies have been conducted in populations of European origin. However, each of the models studied in this research has a high prognostic value and can be used in the future for replicative studies and the development of promising early diagnostic systems based on the individual genetic profile. Nevertheless, the observed relationship between genotypes and continuous traits was largely linear, making methods based on polygenic risk assessment and regression analysis potential avenues for early diagnosis.

A limitation of the study. A limitation related to our study is the small sample size, the number of SNPS studied and the lack of inclusion of clinical predictors in the model studied. In addition, the results have not been tested on other cohorts from the Volga-Ural region. However, the main goal of the study was to develop a polygenic risk scale specifically for the study population of women from the Volga-Ural region who participated in the full genome-wide GWAS study and to understand the potential applicability of this method in the future for early diagnosis of osteoporosis. In the future, we plan to expand the sample with a polygenic risk assessment and to perform this analysis on other cohorts of patients with postmenopausal osteoporosis.

## 4. Methods and Materials

### 4.1. Phenotypic Information

The case-control study involved 701 postmenopausal women (mean age = 61.95 ± 7.94), who underwent medical examination between 2004 and 2011 and lived in the regions of Ekaterinburg and Ufa, Russia. There were women with primary osteoporosis and age-matched control groups without fractures and with a normal level of BMD. The study included 294 women with fractures and 407 without fractures. BMD level was measured in 496 women, including the femoral neck in 270 women and the lumbar spine in 281 women. We completely excluded the presence of any family groups and relatives in the sample on the basis of questionnaires and family history data. The clinical characteristics of the groups are presented in Table 1. Exclusion criteria comprised current use of bisphosphonates or other medications known to affect bone metabolism, a history of alcohol and drug abuse, chronic glucocorticoid use, hormone replacement therapy, current treatment for acute medical condition, bilateral knee and/or hip replacement, metal hardware in both lower extremities, history of heterotopic ossification at the hip and knee, history of cardiovascular disease, besides a history of diseases known to affect bone metabolism (e.g., ankylosing spondylitis, rheumatoid arthritis, Paget’s disease, hyperparathyroidism, hypogonadism, hypothyroidism, diabetes mellitus and/or cancer). The BMD level was measured by biphasic absorption X-ray densitometry (DEXA) using a Hologic QDR 4500/A DXA system (USA) in standard localisations (femoral neck and lumbar spine). The general sample was divided according to T-criterion–a T-value of more than −1.0 described a normal BMD, values from −1.0 to −2.5 diagnosed osteopenia, whilst values less than −2.5 diagnosed osteoporosis (in accordance with the WHO recommendations) [1], and also according to the presence or absence of osteoporetic fractures in standard localisations (axial femur, lumbar spine). Each participant signed an informed consent form to participate in the study, in accordance with the standards developed by the World Medical Association Declaration of Helsinki “Ethical Principles for Scientific Medical Research Involving Human Subjects” and with the approval of the local Bioethics Committee of the Institute of Biochemistry and Genetics UFRC RAS.

### 4.2. Genetic Data, Quality Control and Analysis

The study included 140 polymorphic variants and GWAS replication results within GEFOS (Genetic Factors in Osteoporosis Consortium) [8] and was based on the findings (genotypes and their effects) from previous genetic studies of osteoporosis in the VUR (Supplementary Data), including polymorphic variants of structural genes, transcription factors, steroid hormone receptors, signalling pathways, enzymes, intergenic sites, microRNA genes and their binding sites in addition to other functional regions (Tables S1–S4). Genotyping of the studied loci was conducted using polymerase chain reaction (PCR) “in real time” by way of using TaqMan technology on the platform of the CFX96 device (Biorad Touch Real-Time PCR Detection System).

### 4.3. Statistical Analysis

Genetic heterogeneity of the studied sample was assessed using principal component analysis (PC) in the Smartpca software [36]. Heterogeneity was assessed using the Cochran Q-criterion, differences were considered statistically significant at *p* < 0.1, heterogeneity level was revealed using I^2^ criterion and filtering was performed using PLINK 1.09 software (Cambridge, MA, USA) [37]. Linkage disequilibrium analysis and Hardy-Weinberg equilibrium compliance (*p* < 0.05) were completed using Haploview 4.2 software (Cambridge, MA, USA) [38]. PGS was undertaken using PLINK 1.09 software and was calculated by summing up the effects of risk alleles in everyone, weighted by the effect size of the risk alleles (OR). PGSs obtained for each individual were further normalised using the normalisation script of the R-studio software version 1.4.1717 (Boston, MA, USA). 

The formula for calculating PGS is as follows [39]:

$$PRS=\frac{\sum_{i}^{N}S_{i}*G_{ij}}{P*M_{j}}$$

where *Sj* is the effect size (OR); *Gij* is the observed number of risk alleles; *P* is the sampling density (2 for humans); *N* is the total number of polymorphic variants; *Mj* is the number of missing genotypes in the analysed array.

The PGS assessment included distinctive five steps:

1. Quality control of baseline data with the exclusion of polymorphic loci that are linkage disequilibrium and do not support the Hardy-Weinberg equilibrium.
2. Analysis of polygenic risk assessment.
3. PGS specificity and sensitivity analysis using ROC-analysis.
4. The search for the most sensitive and specific model of polygenic fracture risk and low BMD was completed on the basis of ROC-analysis on the basis of RStudio, in which the sensitivity and specificity of the model as well as the AUC, a numerical index of the area under the ROC-curve, were assessed.

## 5. Conclusions

In conclusion, we generated a polygenic risk score for primary osteoporosis that is consistently associated with fracture risk and low BMD in postmenopausal women from the Volga-Ural region of Russia. By analysing the 3 prognostic models, we found that the model including women with fractures combined with low BMD had the highest sensitivity and specificity. The loci included in the models for analysis are of interest for further research. PGS should be studied as a tool to provide better risk stratification to identify individuals at high risk of fracture, especially given that polygenic risk estimates can be derived from effective early diagnostic methods.

## Figures and Tables

**Table 1:** Characteristics of the study by phenotype subgroups.

Parameters	Woman		
	N	Age, Years, Me±SD	$\mathrm{BMI},\;\mathrm{kg}/m^{2},\;Me\;\pm \;\mathrm{SD}$
With fractures	294	62.16 ± 7.95	27.00 ± 3.60
Without fractures	407	60.14 ± 8.01	27.80 ± 3.81
With low BMD	324	62.17 ± 7.95	27.04 ± 3.60
With normal BMD	172	60.23 ± 7.97	28.10 ± 4.90
With low BMD and with fractures	177	60.15 ± 7.43	27.00 ± 2.40
With normal BMD and without fracture	260	61.76 ± 7.96	28.90 ± 3.70

Abbreviations: Me—mean value, SD—standard deviation.

## Data Availability

The additional data are available in Supplementary.

## Supplementary Materials

The following supporting information can be downloaded at: https://www.mdpi.com/article/10.3390/ijms231710021/s1.
