# Supplementary material for: Using a Polygenic Score to Predict the Risk of Developing Primary Osteoporosis
Source: Int J Mol Sci. 2022 Sep 2;23(17):10021. doi: 10.3390/ijms231710021 (PMC9456390; doi:10.3390/ijms231710021)
Supplement: Supplementary file 1 [file ijms-23-10021-s001.zip › ijms-1831813-supplementary-Table S1.pdf]

Table S1. Characteristic of studied loci from the GEFOS Consortium's GWAS replication as part of a study of a sample of women from the Volga-Ural region of Russia

№	Polymorphic loci ID	Chromosome	Genomic position
1	rs2295294	1	10130826
2	rs7417366	1	168494349
3	rs13464	17	16030775
4	rs10510373	3	7869340
5	rs4832734	4	37701376
6	rs6830890	4	111597970
7	rs17284960	5	164196496
8	rs28425	21	41372958
9	rs1405534	8	25152611
10	rs10756362	9	12344883
11	rs11788458	9	111855741
12	rs13734	20	17614084
13	rs6231	5	96421888
14	rs1282108	5	101445219
15	rs129333	22	36757245
16	rs1048146	16	90043663
17	rs2717096	18	77217086
18	rs1514348	6	151861180
19	rs3020314	6	151949537
20	rs1062033	15	51255741
21	rs28757190	15	51215771
22	rs1801725	3	122284910
23	rs1801197	7	93426441
24	rs182549	2	135859184
25	rs9630182	11	13598625
26	rs7125774	11	13597257
27	rs10518716	15	67202485
28	rs17054320	6	150841717
29	rs2228570	12	47879112
30	rs1544410	12	47846052
31	rs1107946	17	50203629
32	rs2412298	17	50203294
33	rs180012	6	16309435
34	rs545382	11	68403545

35	rs2277268	11	68406654
36	rs9340799	6	151842246
37	rs2120461	1	8387662
38	rs7521902	1	22164231
39	rs6426749	1	22384980
40	rs12137389	1	45472714
41	rs12407028	1	68182033
42	rs11809524	1	102993981
43	rs479336	1	172230433
44	rs12120297	1	223377942
45	rs13413210	2	29349779
46	rs7584262	2	42023409
47	rs4233949	2	54432570
48	rs730402	2	59867571
49	rs17040773	2	111742458
50	rs1878526	2	118281022
51	rs11675051	2	190581704
52	rs12995369	2	201962257
53	rs6436440	2	223840189
54	rs2291296	3	25384391
55	rs7427438	3	29353234
56	rs430727	3	41087073
57	rs1026364	3	113651163
58	rs3755955	4	1000626
59	rs6532023	4	87852697
60	rs1366594	5	89080244
61	rs4957742	5	105837267
62	rs9466056	6	21384382
63	rs11755164	6	44671447
64	rs13204965	6	126845927
65	rs4869742	6	151586613
66	rs7751941	6	151625523
67	rs7788807	7	4728407
68	rs6959212	7	38088724
69	rs2282930	7	50686982
70	rs4727338	7	96491363
71	rs13245690	7	121145010

72	rs3801387	7	121334711
73	rs1670346	7	158474569
74	rs7017914	8	70678968
75	rs13272568	8	78122224
76	rs2062377	8	118995181
77	rs4240467	9	121548011
78	rs7851693	9	130603440
79	rs3905706	10	28191013
80	rs7071206	10	77641558
81	rs2784767	10	80134738
82	rs7084921	10	100054045
83	rs11602954	11	202856
84	rs7108738	11	15688538
85	rs10835187	11	27484130
86	rs163879	11	30930127
87	rs7932354	11	46700671
88	rs3736228	11	68433827
89	rs2887571	12	1529005
90	rs11048046	12	25451875
91	rs7953528	12	27864226
92	rs2016266	12	53334171
93	rs736825	12	54023792
94	rs1053051	12	106973447
95	rs7326472	13	42405815
96	rs1286083	14	90976435
97	rs11623869	14	103417296
98	rs2118784	15	51158988
99	rs9921222	16	325782
100	rs13336428	16	1482462
101	rs4985155	16	15035602
102	rs1564981	16	50952397
103	rs1566045	16	50987892
104	rs4790881	17	2165638
105	rs4792909	17	43721456
106	rs227584	17	44148179
107	rs1864325	17	45900461
108	rs7226305	17	54032130

109	rs7217932	17	71952875
110	rs4796995	18	13708575
111	rs884205	18	62387624
112	rs7257450	19	17377798
113	rs10416218	19	33108221
114	rs3790160	20	10659340
115	rs4817775	21	36112764
116	rs4820539	22	23135783
117	rs5934507	X	8949165
118	rs5926033	X	22666244
119	rs5952638	X	44835659
120	rs4492531	X	75812296
121	rs964181	X	151873604
