# Supplementary material for: Using a Polygenic Score to Predict the Risk of Developing Primary Osteoporosis
Source: Int J Mol Sci. 2022 Sep 2;23(17):10021. doi: 10.3390/ijms231710021 (PMC9456390; doi:10.3390/ijms231710021)
Supplement: Supplementary file 1 [file ijms-23-10021-s001.zip › ijms-1831813-supplementary-Table S2.pdf]

Table S2. Characteristics of the studied polymorphic variants included in the fracture prediction model

SNP	Risk allele	ln OR	OR
rs2120461	T	0,130	1,139
rs2295294	A	0,007	1,007
rs7521902	A	0,145	1,156
rs6426749	G	0,025	1,025
rs12137389	C	0,324	1,383
rs12407028	C	0,016	1,016
rs1031820	A	0,144	1,155
rs11809524	T	0,326	1,386
rs7417366	A	0,029	1,029
rs479336	T	0,123	1,131
rs12120297	C	0,008	1,008
rs13413210	C	0,232	1,261
rs7584262	C	0,115	1,122
rs4233949	G	0,059	1,061
rs730402	G	0,031	1,032
rs17040773	A	0,265	1,304
rs1878526	A	0,212	1,236
rs182549	A	0,207	1,230
rs11675051	A	0,026	1,026
rs12995369	A	0,050	1,051
rs6436440	A	0,052	1,053
rs10510373	G	0,415	1,515
rs2291296	G	0,028	1,028
rs7427438	C	0,031	1,031
rs430727	T	0,017	1,017
rs1026364	G	0,043	1,044
rs1801725	T	0,105	1,111
rs344081	T	0,173	1,189
rs3755955	G	0,051	1,052
rs4832734	T	0,157	1,170
rs6532023	T	0,251	1,285
rs6830890	A	0,099	1,104
rs6854081	G	0,130	1,139
rs1366594	C	0,006	1,006
rs6231	A	0,038	1,039
rs1282108	C	0,021	1,021
rs4957742	A	0,136	1,146
rs1054204	G	0,010	1,010
rs2910164	G	0,100	1,105
rs17284960	C	0,265	1,304
rs180012	T	0,116	1,123
rs9466056	G	0,021	1,021

rs11755164	C	0,077	1,080
rs13204965	A	0,151	1,163
rs2745426	T	0,026	1,026
rs17054320	T	0,262	1,299
rs4869742	T	0,149	1,161
rs7751941	G	0,012	1,012
rs9340799	T	0,114	1,121
rs1514348	G	0,069	1,071
rs3020314	C	0,034	1,035
rs1712	T	0,011	1,011
rs7788807	C	0,268	1,308
rs10226308	A	0,000	1,000
rs6959212	C	0,040	1,041
rs2282930	A	0,028	1,028
rs1801197	C	0,267	1,306
rs4727338	G	0,075	1,078
rs13245690	G	0,156	1,169
rs3801387	G	0,027	1,027
rs7812088	G	0,086	1,090
rs1670346	A	0,205	1,228
rs1405534	C	0,018	1,018
rs7017914	G	0,122	1,130
rs13272568	C	0,209	1,233
rs7844539	A	0,084	1,088
rs3102734	T	0,413	1,511
rs2073618	C	0,060	1,062
rs2062377	T	0,010	1,010
rs10756362	G	0,091	1,095
rs11788458	G	0,039	1,040
rs4240467	C	0,200	1,221
rs7851693	G	0,014	1,014
rs3905706	C	0,125	1,133
rs10793442	A	0,234	1,264
rs7071206	C	0,082	1,085
rs2784767	T	0,148	1,159
rs7084921	C	0,093	1,098
rs11602954	A	0,181	1,198
rs7125774	T	0,051	1,052
rs9630182	C	0,040	1,041
rs7108738	T	0,029	1,029
rs10835187	T	0,100	1,105
rs163879	T	0,199	1,220
rs7932354	T	0,074	1,077
rs198470	T	0,337	1,401
rs545382	C	0,259	1,296
rs2277268	A	0,054	1,055

rs3736228	C	0,154	1,166
rs5854	T	0,043	1,044
rs2887571	G	0,211	1,235
rs11048046	A	0,115	1,122
rs7953528	A	0,162	1,176
rs11540149	A	0,320	1,377
rs1544410	G	0,211	1,235
rs2228570	A	0,265	1,303
rs2016266	G	0,043	1,044
rs11614913	T	0,117	1,124
rs736825	C	0,088	1,092
rs1053051	C	0,083	1,087
rs7326472	G	0,218	1,244
rs1286083	C	0,125	1,133
rs11623869	T	0,058	1,060
rs2118784	A	0,018	1,018
rs28757190	C	0,218	1,244
rs1062033	C	0,007	1,007
rs10518716	G	0,110	1,116
rs9921222	C	0,027	1,027
rs13336428	A	0,216	1,241
rs4985155	G	0,029	1,029
rs1564981	A	0,000	1,000
rs1566045	T	0,121	1,129
rs1048146	A	0,038	1,039
rs4790881	A	0,136	1,146
rs13464	G	0,002	1,002
rs4792909	G	0,042	1,043
rs227584	A	0,248	1,282
rs1864325	C	0,081	1,084
rs1061947	C	0,025	1,025
rs2412298	A	0,112	1,119
rs1107946	G	0,271	1,311
rs7226305	C	0,073	1,076
rs7217932	A	0,016	1,016
rs1042673	G	0,070	1,073
rs4796995	A	0,111	1,117
rs884205	A	0,112	1,119
rs2717096	A	0,126	1,134
rs7257450	A	0,086	1,090
rs10416218	T	0,265	1,304
rs3790160	T	0,080	1,083
rs13734	T	0,228	1,256
rs4817775	C	0,022	1,022
rs28425	T	0,347	1,415
rs4820539	A	0,086	1,090

rs129333	C	0,146	1,157
rs5934507	A	0,075	1,078
rs5926033	C	0,412	1,510
rs5952638	A	0,644	1,904
rs4492531	A	0,016	1,016
rs964181	C	0,135	1,145
