# Supplementary material for: Using a Polygenic Score to Predict the Risk of Developing Primary Osteoporosis
Source: Int J Mol Sci. 2022 Sep 2;23(17):10021. doi: 10.3390/ijms231710021 (PMC9456390; doi:10.3390/ijms231710021)
Supplement: Supplementary file 1 [file ijms-23-10021-s001.zip › ijms-1831813-supplementary-Table S3.pdf]

Table S3. Characteristics of the studied polymorphic variants included in the fracture prediction model

SNP	Risk allele	ln OR	OR
rs2120461	T	0,076	1,079
rs2295294	T	0,166	1,180
rs7521902	A	0,101	1,106
rs6426749	G	0,299	1,348
rs12137389	C	0,631	1,879
rs12407028	T	0,119	1,126
rs1031820	G	0,234	1,264
rs11809524	T	0,364	1,439
rs7417366	G	0,083	1,087
rs479336	G	0,010	1,010
rs12120297	C	0,264	1,302
rs13413210	A	0,122	1,130
rs7584262	T	0,094	1,099
rs4233949	G	0,291	1,338
rs730402	A	0,266	1,305
rs17040773	A	0,031	1,032
rs1878526	A	0,203	1,225
rs182549	A	0,166	1,180
rs11675051	A	0,112	1,118
rs12995369	G	0,201	1,223
rs6436440	G	0,028	1,028
rs10510373	G	0,077	1,080
rs2291296	A	0,099	1,104
rs7427438	A	0,333	1,395
rs430727	C	0,053	1,054
rs1026364	G	0,037	1,038
rs1801725	T	0,377	1,458
rs344081	C	0,081	1,084
rs3755955	A	0,329	1,389
rs4832734	T	0,163	1,177
rs6532023	T	0,059	1,061
rs6830890	G	0,089	1,093
rs6854081	G	0,146	1,157
rs1366594	C	0,114	1,121
rs6231	A	0,015	1,015
rs1282108	T	0,078	1,081
rs4957742	A	0,128	1,136
rs1054204	G	0,005	1,005
rs2910164	C	0,186	1,204
rs17284960	T	0,231	1,260
rs180012	G	0,012	1,012
rs9466056	A	0,220	1,246
rs11755164	T	0,103	1,108
rs13204965	A	0,085	1,089
rs2745426	T	0,217	1,242

rs17054320	A	0,264	1,302
rs4869742	T	0,075	1,078
rs7751941	G	0,306	1,358
rs9340799	T	0,004	1,004
rs1514348	G	0,141	1,151
rs3020314	C	0,007	1,007
rs1712	C	0,147	1,158
rs7788807	C	0,166	1,181
rs10226308	A	0,209	1,233
rs6959212	C	0,103	1,109
rs2282930	G	0,067	1,069
rs1801197	C	0,048	1,049
rs4727338	G	0,121	1,129
rs13245690	G	0,196	1,217
rs3801387	G	0,093	1,098
rs7812088	G	0,058	1,060
rs1670346	A	0,117	1,124
rs1405534	C	0,189	1,208
rs7017914	A	0,188	1,207
rs13272568	A	0,035	1,036
rs7844539	C	0,215	1,240
rs3102734	C	0,137	1,147
rs2073618	C	0,016	1,016
rs2062377	A	0,162	1,176
rs10756362	G	0,036	1,037
rs11788458	G	0,155	1,168
rs4240467	G	0,038	1,039
rs7851693	G	0,075	1,078
rs3905706	T	0,152	1,164
rs10793442	A	0,303	1,354
rs7071206	T	0,083	1,087
rs2784767	T	0,135	1,144
rs7084921	C	0,098	1,103
rs11602954	A	0,250	1,284
rs7125774	T	0,065	1,067
rs9630182	T	0,044	1,045
rs7108738	T	0,028	1,028
rs10835187	C	0,094	1,099
rs163879	C	0,107	1,113
rs7932354	C	0,115	1,122
rs198470	T	0,563	1,756
rs545382	C	0,280	1,323
rs2277268	A	0,662	1,938
rs3736228	T	0,050	1,051
rs5854	T	0,107	1,113
rs2887571	G	0,243	1,275
rs11048046	A	0,123	1,131
rs7953528	A	0,022	1,022
rs11540149	A	0,240	1,271

rs1544410	A	0,142	1,153
rs2228570	A	0,139	1,149
rs2016266	G	0,118	1,125
rs11614913	T	0,156	1,169
rs736825	G	0,049	1,050
rs1053051	C	0,060	1,062
rs7326472	G	0,338	1,402
rs1286083	C	0,322	1,380
rs11623869	T	0,075	1,078
rs2118784	A	0,182	1,200
rs28757190	C	1,161	3,193
rs1062033	G	0,005	1,005
rs10518716	C	0,177	1,194
rs9921222	T	0,012	1,012
rs13336428	G	0,235	1,265
rs4985155	G	0,060	1,062
rs1564981	G	0,182	1,200
rs1566045	T	0,351	1,420
rs1048146	A	0,005	1,005
rs4790881	C	0,162	1,176
rs13464	A	0,134	1,143
rs4792909	G	0,213	1,238
rs227584	C	0,036	1,037
rs1864325	T	0,031	1,031
rs1061947	C	0,200	1,221
rs2412298	G	0,011	1,011
rs1107946	G	0,138	1,148
rs7226305	C	0,163	1,177
rs7217932	G	0,075	1,078
rs1042673	A	0,106	1,112
rs4796995	A	0,293	1,340
rs884205	C	0,101	1,106
rs2717096	G	0,204	1,226
rs7257450	A	0,003	1,003
rs10416218	C	0,098	1,103
rs3790160	C	0,221	1,247
rs13734	T	0,136	1,146
rs4817775	C	0,154	1,166
rs28425	G	0,812	2,252
rs4820539	G	0,170	1,185
rs129333	C	0,501	1,650
rs5934507	G	0,152	1,164
rs5926033	C	0,289	1,335
rs5952638	A	0,114	1,121
rs4492531	A	0,044	1,045
rs964181	C	0,224	1,251
