# Supplementary material for: Using a Polygenic Score to Predict the Risk of Developing Primary Osteoporosis
Source: Int J Mol Sci. 2022 Sep 2;23(17):10021. doi: 10.3390/ijms231710021 (PMC9456390; doi:10.3390/ijms231710021)
Supplement: Supplementary file 1 [file ijms-23-10021-s001.zip › ijms-1831813-supplementary-Table S4.pdf]

Table S4. Characteristics of the studied polymorphic variants included in the fracture prediction model at a low level of BMD

SNP	Risk allele	ln OR	OR
rs2120461	T	0,241	1,272
rs2295294	T	0,218	1,243
rs7521902	A	0,188	1,207
rs6426749	G	0,226	1,253
rs12137389	C	0,652	1,920
rs12407028	T	0,094	1,099
rs1031820	G	0,183	1,201
rs11809524	T	0,784	2,191
rs7417366	G	0,072	1,075
rs479336	T	0,088	1,092
rs12120297	C	0,321	1,378
rs13413210	A	0,078	1,081
rs7584262	C	0,075	1,078
rs4233949	G	0,256	1,292
rs730402	A	0,204	1,226
rs17040773	A	0,219	1,245
rs1878526	A	0,267	1,306
rs182549	A	0,268	1,307
rs11675051	A	0,131	1,140
rs12995369	G	0,105	1,111
rs6436440	A	0,108	1,114
rs10510373	G	0,333	1,395
rs2291296	A	0,186	1,204
rs7427438	A	0,242	1,274
rs430727	C	0,015	1,015
rs1026364	G	0,075	1,078
rs1801725	T	0,435	1,545
rs344081	T	0,164	1,178
rs3755955	A	0,357	1,429
rs4832734	T	0,191	1,211
rs6532023	T	0,293	1,340
rs6830890	A	0,005	1,005
rs6854081	G	0,275	1,316
rs1366594	C	0,214	1,239
rs6231	A	0,045	1,046
rs1282108	C	0,038	1,039
rs4957742	A	0,181	1,199
rs1054204	G	0,004	1,004
rs2910164	C	0,063	1,065
rs17284960	C	0,066	1,068
rs180012	G	0,024	1,024
rs9466056	A	0,141	1,151
rs11755164	T	0,018	1,018
rs13204965	A	0,304	1,355
rs2745426	T	0,218	1,243

rs17054320	A	0,100	1,105
rs4869742	T	0,284	1,328
rs7751941	G	0,322	1,380
rs9340799	T	0,057	1,059
rs1514348	G	0,113	1,120
rs3020314	C	0,006	1,006
rs1712	C	0,073	1,076
rs7788807	C	0,341	1,407
rs10226308	A	0,176	1,192
rs6959212	C	0,240	1,271
rs2282930	A	0,071	1,074
rs1801197	C	0,165	1,179
rs4727338	G	0,112	1,119
rs13245690	G	0,305	1,357
rs3801387	G	0,021	1,021
rs7812088	G	0,127	1,135
rs1670346	A	0,264	1,302
rs1405534	C	0,208	1,231
rs7017914	A	0,094	1,099
rs13272568	C	0,093	1,098
rs7844539	C	0,108	1,114
rs3102734	T	0,307	1,360
rs2073618	C	0,126	1,134
rs2062377	A	0,108	1,114
rs10756362	G	0,020	1,020
rs11788458	G	0,125	1,133
rs4240467	C	0,256	1,292
rs7851693	C	0,085	1,089
rs3905706	C	0,139	1,149
rs10793442	A	0,420	1,522
rs7071206	T	0,057	1,059
rs2784767	T	0,255	1,290
rs7084921	C	0,196	1,216
rs11602954	A	0,311	1,365
rs7125774	T	0,117	1,124
rs9630182	C	0,067	1,069
rs7108738	T	0,020	1,020
rs10835187	T	0,017	1,017
rs163879	C	0,031	1,032
rs7932354	C	0,021	1,021
rs198470	T	0,984	2,676
rs545382	C	0,480	1,616
rs2277268	A	0,878	2,406
rs3736228	T	0,039	1,040
rs5854	T	0,136	1,146
rs2887571	G	0,397	1,487
rs11048046	A	0,383	1,466
rs7953528	A	0,211	1,235
rs11540149	A	0,514	1,672

rs1544410	A	0,004	1,004
rs2228570	A	0,389	1,475
rs2016266	G	0,096	1,101
rs11614913	T	0,262	1,299
rs736825	G	0,007	1,007
rs1053051	C	0,118	1,125
rs7326472	G	0,384	1,468
rs1286083	C	0,358	1,430
rs11623869	T	0,101	1,106
rs2118784	A	0,136	1,146
rs28757190	C	1,299	3,667
rs1062033	G	0,000	1,000
rs10518716	C	0,174	1,190
rs9921222	T	0,012	1,012
rs13336428	G	0,063	1,065
rs4985155	G	0,189	1,208
rs1564981	G	0,096	1,101
rs1566045	T	0,229	1,257
rs1048146	A	0,085	1,089
rs4790881	A	0,030	1,030
rs13464	A	0,146	1,157
rs4792909	G	0,172	1,188
rs227584	A	0,145	1,156
rs1864325	T	0,002	1,002
rs1061947	C	0,214	1,239
rs2412298	A	0,033	1,034
rs1107946	G	0,300	1,350
rs7226305	C	0,196	1,217
rs7217932	G	0,019	1,019
rs1042673	G	0,081	1,084
rs4796995	A	0,387	1,472
rs884205	C	0,051	1,052
rs2717096	G	0,237	1,268
rs7257450	G	0,110	1,116
rs10416218	T	0,034	1,035
rs3790160	C	0,059	1,061
rs13734	T	0,293	1,341
rs4817775	C	0,160	1,174
rs28425	G	0,582	1,790
rs4820539	A	0,003	1,003
rs129333	C	0,476	1,610
rs5934507	A	0,100	1,105
rs5926033	C	0,588	1,800
rs5952638	A	0,504	1,655
rs4492531	A	0,187	1,206
rs964181	C	0,287	1,332
